# Micro-epidemiology of mixed-species malaria infections in a rural population living in the Colombian Amazon region

**DOI:** 10.1038/s41598-018-23801-9

**Published:** 2018-04-03

**Authors:** Milena Camargo, Sara C. Soto-De León, Luisa Del Río-Ospina, Astrid C. Páez, Zanony González, Edgardo González, Juan R. Cubides, Paola A. Camargo-Ayala, Manuel E. Patarroyo, Manuel A. Patarroyo

**Affiliations:** 1 Molecular Biology and Immunology Department, Fundación Instituto de Inmunología de Colombia (FIDIC), Bogotá, Colombia; 2 PhD Programme in Biomedical and Biological Sciences, School of Medicine and Health Sciences, Universidad del Rosario, Bogotá, DC Colombia; 3 Universidad de Ciencias Aplicadas y Ambientales (UDCA), Bogotá, Colombia; 4 School of Medicine, Universidad Nacional de Colombia, Bogotá, Colombia; 5 Masters in Epidemiology Programme, School of Medicine and Health Sciences, Universidad del Rosario, Bogotá, DC Colombia; 6 Basic Sciences Department, School of Medicine and Health Sciences, Universidad del Rosario, Bogotá, Colombia

## Abstract

Malaria outbreaks have been reported in recent years in the Colombian Amazon region, malaria has been re-emerging in areas where it was previously controlled. Information from malaria transmission networks and knowledge about the population characteristics influencing the dispersal of parasite species is limited. This study aimed to determine the distribution patterns of *Plasmodium vivax, P. malariae* and *P. falciparum* single and mixed infections, as well as the significant socio-spatial groupings relating to the appearance of such infections. An active search in 57 localities resulted in 2,106 symptomatic patients being enrolled. Parasitaemia levels were assessed by optical microscopy, and parasites were detected by PCR. The association between mixed infections (in 43.2% of the population) and socio-spatial factors was modelled using logistic regression and multiple correspondence analyses. *P. vivax* occurred most frequently (71.0%), followed by *P. malariae* (43.2%), in all localities. The results suggest that a parasite density-dependent regulation model (with fever playing a central role) was appropriate for modelling the frequency of mixed species infections in this population. This study highlights the under-reporting of *Plasmodium* spp. mixed infections in the malaria-endemic area of the Colombian Amazon region and the association between causative and environmental factors in such areas.

## Introduction

Malaria is considered as the parasitic disease that has the greatest impact on public health^1^. *Plasmodium* spp. infection becomes perpetuated in a cycle of disease and poverty, contributing towards affected individuals’ worsening quality of life and limiting the possibility of eradicating such infections^2^.

Malaria is transmitted by female mosquitoes from the genus *Anopheles*, with mammals being the definitive host^1,3^. Six species from the genus *Plasmodium* have been described as causing malaria in human beings: *P. falciparum, P. vivax, P. malariae, P. ovale curtisi, P. ovale wallikeri* and *P. knowlesi*^4,5^, with *Plasmodium* spp. being endemic in 91 countries and causing 212 million cases of infection per year (429,000 leading to death)^6^.

Current mitigation measures in disease-endemic countries have not had the desired impact since an increase in malaria cases has been reported for countries such as Colombia, where 55,866 cases were confirmed in 2015 (annual parasite index: 5.4 cases per 1,000 inhabitants)^7,8^. Colombia thus accounts for 10% of cases of malaria in the Americas^9–11^, with Colombia’s Amazon region being the focus of an outbreak of malaria during the last few years^7,12^.

The Amazon basin covering a large part of southern Colombia (108,951 km^2^) is a major transmission and disease load foci^13,14^, which operates relatively independently from other Colombian regions. The Amazon region’s habitat diversity and its own climatic characteristics (seasonal rainfall effects) determine vector presence and abundance (i.e. *Anopheles benarrochi, Anopheles oswaldoi, Anopheles darlingi*). Such vectors are anthropophilic and highly efficient regarding parasite transmission and have facilitated the increase in cases of malaria amongst the region’s inhabitants, together with the demographics of human settlements, and clinical and housing conditions in the region and their related dynamics^14,15^.

Risk factors for acquiring malaria have been described on different levels (genetic, social determinants and environmental) and influence exposure to parasitic infection, its course and outcomes^16–19^. These factors also facilitate infection by more than one *Plasmodium* spp. (mixed-species malaria); however, these mixed-species are currently being under-diagnosed given the use of conventional techniques^10^. Little is currently known regarding the biology and establishment of *Plasmodium* mixed infections, but insight into the frequency of mixed-species infections in the population and the factors affecting their transmission is essential for developing effective disease elimination measures^20,21^. The factors involved in malaria transmission and those influencing mixed *Plasmodium* spp. species infection in highly endemic regions need to be determined, particularly at a time when rapid climatic changes can modify host-vector-pathogen relationship dynamics.

This study aimed to establish the frequency of three *Plasmodium* spp. within the population, determine the distribution of mixed infections and identify infected patient profiles in the Colombian Amazon region.

## Results

### Characteristics of the population being analysed

**Figure 1:**
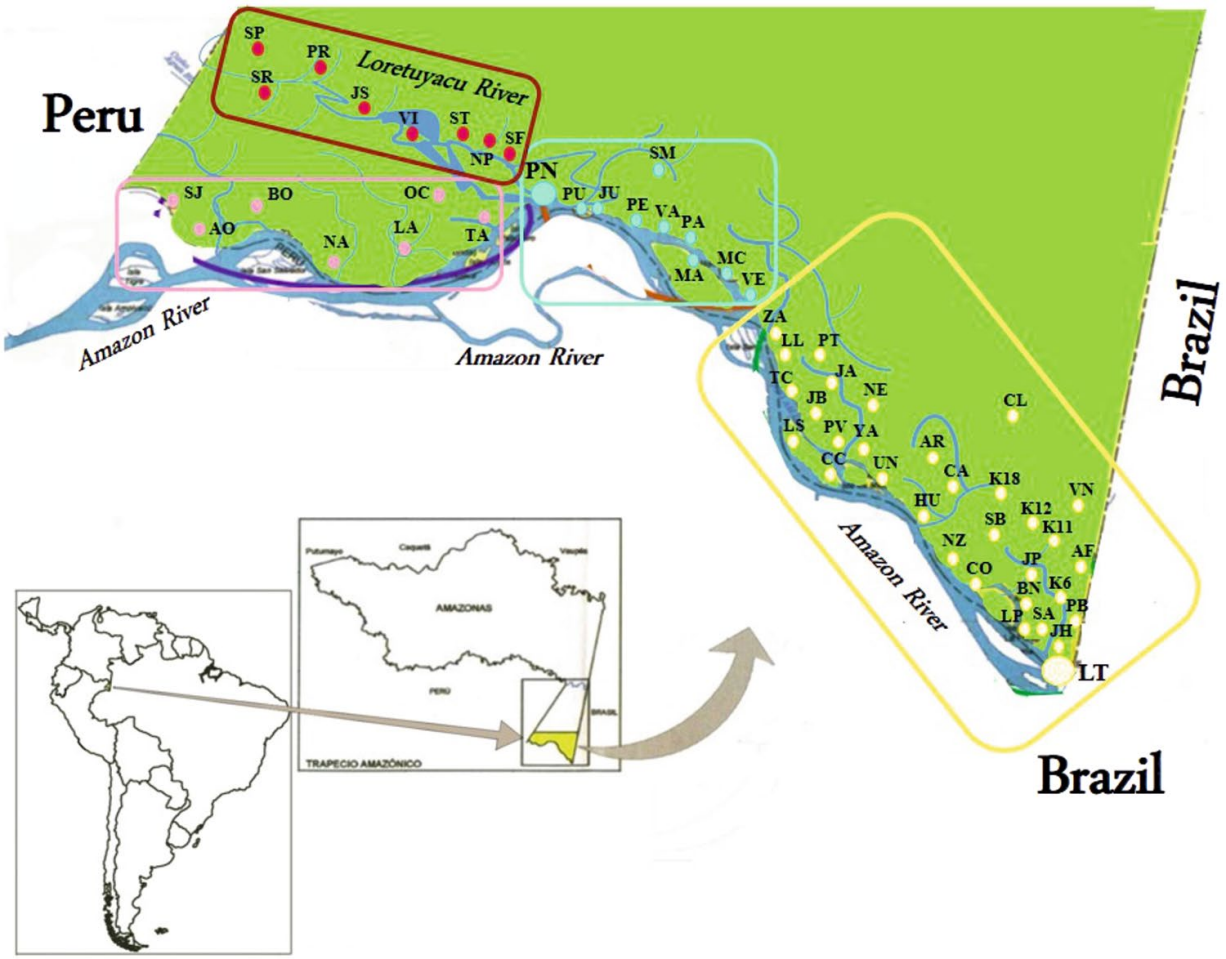
Geographical locations of the 57 localities where samples were collected (this map was modified from a map downloaded from the Instituto Geográfico Agustín Codazzi, IGAC)^60,61^. Images are freely accessible and modifiable in accordance with IGAC policies.

Of the 2,106 patients invited to participate in the study, 5.3% (n = 111) were excluded due to negative results with human *β-globin* gene amplification; 1,995 subjects thus became the object of statistical analysis. The sampling region was divided into areas in accordance with the population characteristics (Fig. 1); 344 samples were taken in area 1, 257 samples in area 2, 566 samples in area 3 and 828 samples in area 4 (Additional file 1: Table S1). The average age of the population was 26.6 years (SD: 19.8 years) and 48.2% (n = 961) reported a previous episode of malaria, mainly those living in area 4 (n = 441). Table 1 provides the distribution of sociodemographic characteristics amongst the population in accordance with the *Plasmodium* spp. infection stage (as determined by molecular biology).

**Table 1:** Sociodemographic characteristics of the sample population.

Variable	Uninfected (n = 245)		Single infection (n = 906)		Mixed infection (n = 844)		*P. vivax* (n = 1,412)		*P. falciparum* (n = 432)		*P. malariae* (n = 862)	
	n	%	n	%	n	%	n	%	n	%	n	%
**Age in years**												
≤5	54	22	138	15.2	112	13.3	190	13.5	67	15.5	118	13.7
6–12	38	15.5	174	19.2	165	19.5	269	19.1	89	20.6	170	19.7
13–18	17	6.9	88	9.7	90	10.7	150	10.6	37	8.6	94	10.9
19–30	51	20.8	164	18.1	152	18	256	18.1	82	19	153	17.7
31–60	75	30.6	275	30.4	263	31.2	436	30.9	121	28	273	31.7
≥60	10	4.1	67	7.4	62	7.3	111	7.9	36	8.3	54	6.3
**Gender**												
Male	125	51	444	49	444	52.6	689	48.8	227	52.5	446	51.7
Female	120	49	462	51	400	47.4	723	51.2	205	47.5	416	48.3
**Sampling area**												
Area 1	49	20	166	18.3	129	15.3	246	17.4	18	4.2	164	19
Area 2	21	8.6	102	11.3	134	15.9	197	13.9	84	19.4	109	12.7
Area 3	62	25.3	271	29.9	233	27.6	404	28.6	123	28.5	252	29.2
Area 4	113	46.1	367	40.5	348	41.2	565	40	207	47.9	337	39.1
**Settlement type**												
Rural	224	91.4	791	87.3	755	89.5	1,350	95.6	428	99.1	828	96.1
Urban	21	8.6	115	12.7	89	10.5	62	4.4	4	0.9	34	3.9
**Stagnant water nearby**												
No	90	36.7	555	61.3	539	63.9	893	63.2	285	66	533	61.8
Yes	155	63.3	351	38.7	305	36.1	519	36.8	147	34	329	38.2
**Insecticide use**												
No	208	84.9	783	86.4	752	89.1	1,232	87.3	397	91.9	759	88.1
Yes	37	15.1	123	13.6	92	10.9	180	12.7	35	8.1	103	11.9
**Mosquito net use**												
No	13	5.3	53	5.8	48	5.7	83	5.9	17	3.9	52	6
Yes	232	94.7	853	94.2	796	94.3	1,329	94.1	415	96.1	810	94
**Public gas supply**												
No	230	93.9	854	94.3	808	95.7	1,337	94.7	421	97.5	821	95.2
Yes	15	6.1	52	5.7	36	4.3	75	5.3	11	2.5	41	4.8
**Public electricity supply**												
No	18	7.3	78	8.6	100	11.8	147	10.4	39	9	107	12.4
Yes	227	92.7	828	91.4	744	88.2	1,265	89.6	393	91	755	87.6
**Public water supply**												
No	207	84.5	613	67.7	554	65.6	926	65.6	310	71.8	560	65
Yes	38	15.5	293	32.3	290	34.4	486	34.4	122	28.2	302	35
**Sewerage service**												
No	207	89.5	666	78.6	636	79.2	1,039	77.3	347	81.6	641	78.2
Yes	28	10.5	190	21.4	175	20.8	305	22.7	78	78.4	179	21.8

Molecular biology was used for determining the *Plasmodium* infection stage and species.

### Detecting *Plasmodium* spp. by conventional microscopy and PCR

By analysing thick blood smears (TBS), 37% (n = 737/1,995) of the population were identified as positive for *Plasmodium* spp., 31.3% (n = 625/1,995) for *P. vivax*, 6.4% (n = 128/1,995) for *P. falciparum* and less than 1% (n = 16/1,995) had mixed-species infections (Additional file 2: Fig. S1a). Parasitaemia varied from 32 to 85,320 parasites/µL blood (mean: 10,100; SD: 11,603), being higher in *P. vivax* (mean: 10,585; SD: 11,920) than in *P. falciparum* (mean: 7,752; SD: 9,099).

**Figure 2:**
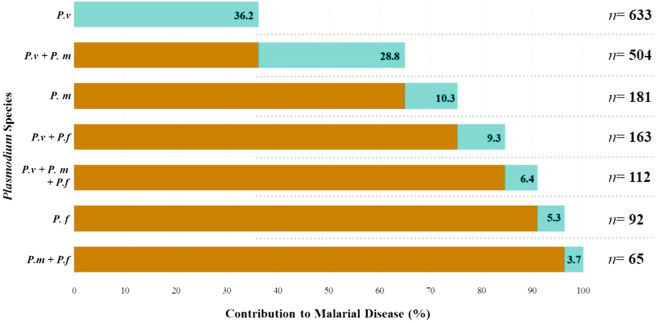
Cumulative frequency of *Plasmodium* species and their contribution to malaria in 1,750 people in whom parasitic DNA was identified using molecular techniques. *P.v* = *Plasmodium vivax*, *P.m* = *Plasmodium malariae* and *P.f* = *Plasmodium falciparum*.

Regarding parasite DNA detection, 88% (n = 1,750/1,995) of the target population were infected with *Plasmodium* spp., with *P. vivax* being the most prevalent species (71.0%; n = 1,412/1,995), followed by *P. malariae* (43.2%; n = 862/1,995) and *P. falciparum* (21.7%; n = 432/1,995). Mixed infection events (simultaneous infection by ≥2 species) were found in 43.2% (n = 844/1,995) of the target population (Additional file 2: Fig. S1b), with the *P. vivax/P. malariae* combination being the most frequently detected (n = 504/1,995) (Fig. 2).

It was found that 75% of the cases were infected with *P. vivax* and *P. malariae* (Fig. 2). Parasite frequency ranged from 82% to 100% (Additional file 3: Fig. S2a) when evaluating parasite infection with respect to age and *P. vivax* was the most prevalent species amongst all age groups, showing a greater frequency in the 31–60-year-old age group (p = 0.002; Chi^2^ tests) (Table 1; Additional file 3: Fig. S2b).

### Evaluating the sampling areas and the types of settlement

**Figure 3:**
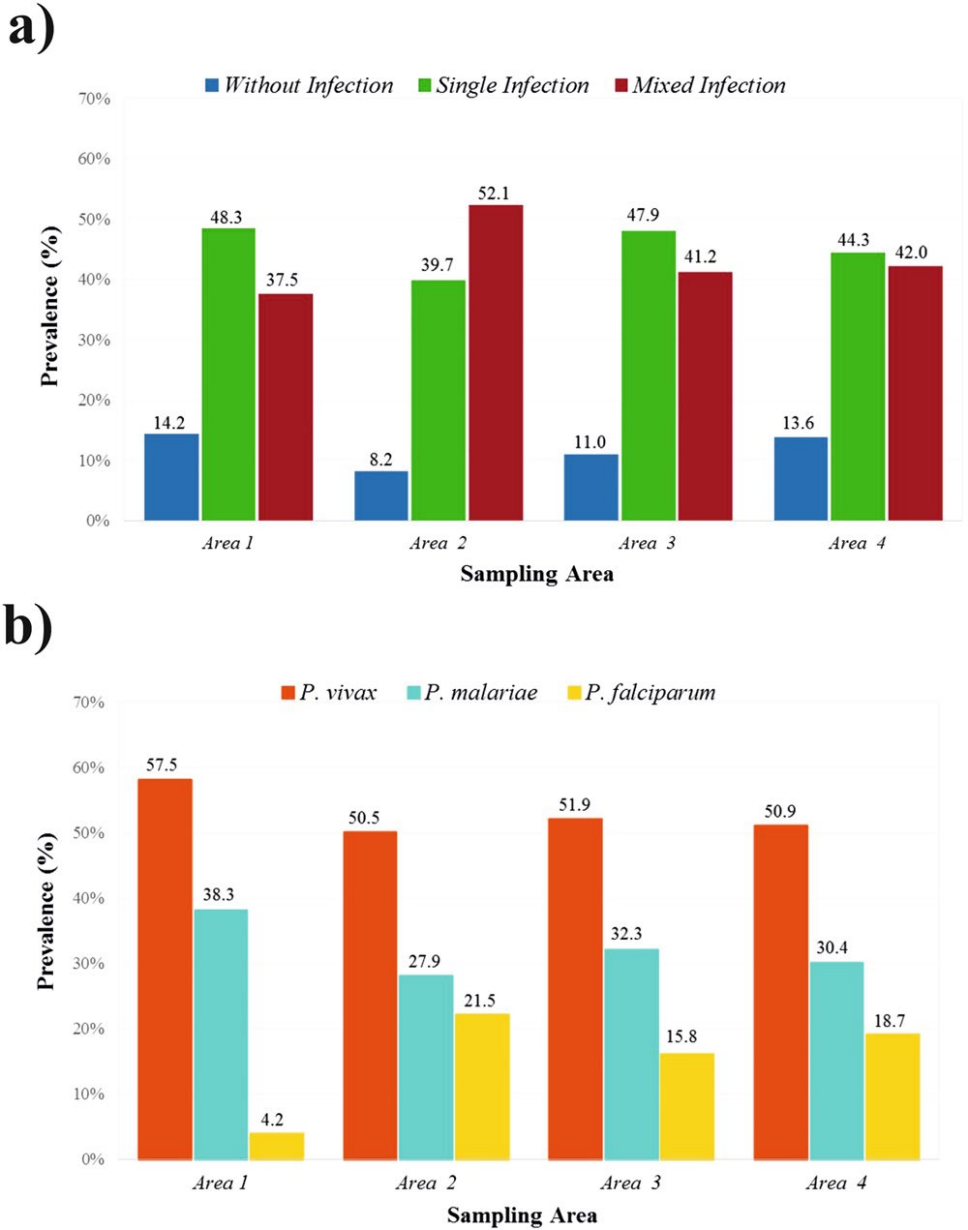
Relative frequency of parasite infection and *Plasmodium* spp. distribution with respect to the area sampled [area 1 (n = 344), area 2 (n = 257), area 3 (n = 566) and area 4 (n = 828)]. Part (**a**) shows the distribution of parasite infection frequency with respect to the *Plasmodium* spp. infection status. Blue represents the uninfected target population. Green represents the proportion of the target population infected by a single species. Dark red represents the proportion of the target population with a mixed species infection. Part (**b**) shows the relative frequency of *Plasmodium* spp.

Additional analysis evaluated the parasite infection status (single and mixed), the mean rate of *Plasmodium* spp. parasitaemia and the distribution with respect to the area sampled. Sampling area 1 had the highest single infection frequency (48.3%) (not statistically significant: p = 0.561; Chi^2^ test); mixed infections appeared most frequently in area 2 (p = 0.001; Chi^2^ test) (Fig. 3a). Mean parasitaemia levels were lower in cases of single infection (9,854 parasites/µL) than in mixed infections (10,394 parasites/µL), but this difference was not statistically significant (p = 0.533; T-test) (Additional file 4: Fig. S3a). However, parasitaemia varied significantly depending on the area being sampled (p = 0.026; ANOVA test). Bonferroni test correction showed significant differences between areas 3 and 4 (p = 0.022) (Additional file 4: Fig. S3b).

The results of the analysis of *Plasmodium* spp. distribution with respect to area showed that *P. vivax* had the greatest frequency (greater than 65%) in almost all localities, except for *P. vivax* in Punta Brava and Yaguas (Additional file 5: Table S3) and *P. malariae* (absent in seven localities evaluated) (Additional file 5: Table S3) (40.7% to 47.7% relative frequencies). *P. falciparum* prevalence was significantly lower in area 1 relative to all other (p = 0.001; Fisher’s exact test) (Fig. 3b).

Parasite infection was evaluated with respect to the type of settlement; Leticia and Puerto Nariño are urban settlements; the remaining localities are rural. There were similar infection percentages for all types of settlement; however, the parasite density index (PDI) was higher for rural areas (index: 57.8). *P. falciparum* infection was mostly restricted to rural settlements (Additional file 6: Table S3).

### *Plasmodium* spp. infection profiles

A clinical profile was created for each participant based on the symptoms reported in a survey conducted during sampling. Vomiting (p = 0.018; Fisher’s exact test) and diarrhoea (p = 0.005; Fisher’s exact test) occurred most frequently in the study population with single *Plasmodium* spp. infections, whereas severe headache was most frequently reported in the population with mixed-species infections (p = 0.001; Fisher’s exact test) (Additional file 7: Fig. S4). The distribution of symptoms was similar for all species of infecting *Plasmodium*, with fever being the most frequently reported symptom amongst the three species (89% to 91%) and a rash being the least frequently reported symptom in the sample population (2.1% to 3.2%) (Additional file 8: Fig. S5).

Logistic regression was used to identify the association between the variables evaluated (age, area, parasitaemia, access to basic services (public water and electricity supply, sewerage service), nearby water stagnations, use of mosquito nets and use of insecticides) and the presence of mixed-species infection. Patients having 2,000 to 4,999 parasites/µL blood parasitaemia [adjusted odds ratio (aOR) 0.61: 0.38–0.98, 95% confidence interval (CI)] or 5,000 to 9,999 parasites/µL blood parasitaemia (aOR 0.48: 0.29–0.77, 95% CI) had a lower probability of acquiring a mixed infection. No significant associations were observed for the other variables included in the model (Table 2).

**Table 2:** Risk factors associated with mixed infections.

**Variable**	**OR Adjusted**	**95% CI**	**p-value**
**Age in years**			
≤5	0.91	0.52–1.59	0.767
6–12	1.11	0.57–2.14	0.749
13–18	1.12	0.62–2.00	0.703
19–30	1.13	0.66–1.95	0.636
31–60	Reference		
≥60	0.60	0.26–1.30	0.237
**Gender**			
Male	Reference		
Female	1.17	0.85–1.61	0.307
**Sampling area**			
Area 1	0.93	0.59–1.48	0.787
Area 2	1.43	0.84–2.48	0.183
Area 3	0.43	0.14–1.30	0.138
Area 4	Reference		
**Stagnant water nearby**			
No	Reference		
Yes	0.98	0.67–1.38	0.926
**Insecticide use**			
No	Reference		
Yes	0.91	0.77–1.07	0.284
**Mosquito net use**			
No	Reference		
Yes	0.77	0.39–1.52	0.463
**Public gas supply**			
No	Reference		
Yes	0.83	0.57–1.20	0.332
**Public electricity supply**			
No	Reference		
Yes	0.81	0.46–1.42	0.472
**Public water supply**			
No	Reference		
Yes	1.55	0.88–2.71	0.122
**Sewerage service**			
No	Reference		
Yes	0.61	0.34–1.11	0.112
**Fever**			
No	Reference		
Yes	0.91	0.41–2.04	0.831
**Parasitaemia**			
1–1,999	0.90	0.58–1.37	0.629
2,000–4,999	**0.61**	**0.38–0.98**	**0.043**
5,000–9,999	**0.48**	**0.29–0.77**	**0.003**
>9,999	Reference		

Values shown in bold p < 0.05. OR adjusted for inhabitants’ age and the urban or rural area in which they reside. Parasitaemia as determined from thick blood smears, and housing conditions, such as the availability of sewerage, drinkable water, gas and electricity services and whether there was stagnant water nearby and whether mosquito nets and insecticides were being used.

Analysing the strength of the association between sociodemographic, clinical and laboratory variables (as previously mentioned), and the combination of parasite species revealed positive associations between area 1 and mixed *P. vivax* and *P. malariae* infections (aOR 2.13: 1.33–3.42, 95% CI), access to a public water supply and mixed *P. malariae* and *P. falciparum* infections (aOR 6.90: 4.98–8.28, 95% CI) and triple infections (simultaneous infection by the three species being evaluated) (aOR 3.05: 1.20–7.74, 95% CI). Variables showing less significant associations were parasitaemia (5,000 to 9,999 parasites/µL blood) in *P. malariae* and *P. falciparum* infections (aOR 0.18: 0.35–0.93, 95% CI) and triple infection events with parasitaemia higher than 2,000 parasites/µL blood and area 1 (Additional file 9: Table S4).

**Figure 4:**
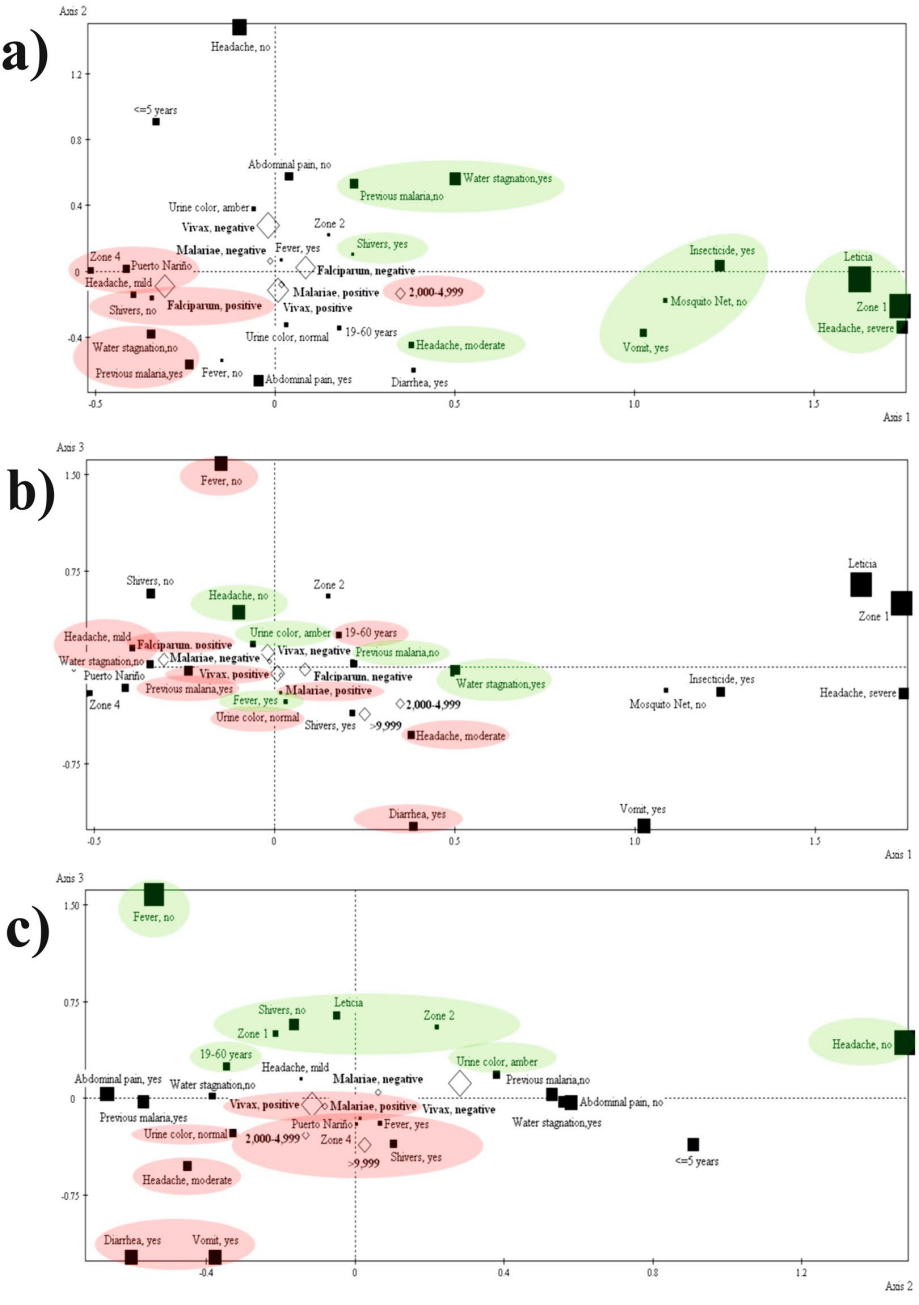
Multiple correspondence analysis (MCA). Part (**a**) represents the mode on axes 1 and 2. Part (**b**) represents the mode on axes 1 and 3. Part (**c**) represents the mode on axes 2 and 3. The variables contributing towards each profile are highlighted; green indicates the positive pole and red indicates the negative pole variables.

Multiple correspondence analysis (MCA) was used for identifying *Plasmodium* spp. infection profiles by compiling clinical and sociodemographic variables (Tables 3 and 4). Three main axes emerged after analysing the change in inertia in the histogram showing the eigenvalues of the active variables (Table 5 and Fig. 4). Three profiles were constructed around these axes (epidemiological and clinical variables related to *P. falciparum* infection, those related to triple infection (*P. falciparum, P. vivax* and *P. malariae*) and those related to double infection by *P. vivax* and *P. malariae*) (Table 5 and Fig. 4).

**Table 3:** Contribution, cosine squared and active variable test values.

Active variables	n	Contribution			Cosine squared			Test value		
		Axis 1	Axis 2	Axis 3	Axis 1	Axis 2	Axis 3	Axis 1	Axis 2	Axis 3
**Age in years**										
≤5	304	0.65	**6.97**	1.44	0.02	**0.15**	0.02	−6.26	**17.21**	−6.89
6–12	377	0.62	1.97	1.57	0.02	0.04	0.03	−6.27	9.35	−7.35
13–18	195	0.24	0.21	0.68	0.01	0.00	0.01	3.65	−2.90	−4.61
19–60	976	0.61	**3.22**	2.13	0.03	**0.11**	0.06	7.83	−**15.07**	10.79
>60	143	0.02	0.82	0.88	0.00	0.02	0.01	−1.13	−5.63	5.16
**Gender**										
Male	1,013	0.02	0.06	0.80	0.00	0.00	0.02	1.54	2.12	−6.73
Female	982	0.02	0.06	0.82	0.00	0.00	0.02	−1.54	−2.12	6.73
**Origin**										
Puerto Nariño	401	**5.29**	0.01	1.49	**0.67**	0.00	0.10	−**36.59**	1.17	−14.30
Leticia	1,591	**20.91**	0.03	**5.89**	**0.67**	0.00	0.10	**36.48**	−1.12	**14.32**
**Area**										
1	344	**20.42**	0.44	**3.04**	**0.63**	0.01	0.05	**35.48**	−4.35	**10.14**
2	257	0.11	0.34	**2.79**	0.00	0.01	0.05	2.55	3.77	**9.47**
3	566	1.56	0.01	1.29	0.06	0.00	0.03	−10.53	0.70	−7.09
4	828	**4.27**	0.00	1.25	**0.19**	0.00	0.03	−**19.30**	0.14	−7.73
**Mosquito net use**										
No	114	**2.64**	0.10	0.13	**0.07**	0.00	0.00	**11.95**	−1.97	−1.95
Yes	1,881	0.16	0.01	0.01	0.07	0.00	0.00	−11.95	1.97	1.95
**Insecticide use**										
No	1,743	1.10	0.00	0.05	0.22	0.00	0.00	−21.05	−0.56	3.28
Yes	252	**7.59**	0.01	0.34	**0.22**	0.00	0.00	**21.05**	0.56	−3.28
**Stagnant water nearby**										
No	1,184	**2.75**	**4.82**	0.02	0.17	**0.21**	0.00	−**18.57**	−**20.68**	1.03
Yes	811	**4.01**	**7.04**	0.02	0.17	**0.21**	0.00	**18.57**	**20.68**	−1.03
**Fever**										
No	223	0.10	1.81	**19.92**	0.00	0.04	**0.31**	−2.34	−8.56	**25.05**
Yes	1,772	0.01	0.23	**2.51**	0.00	0.04	**0.31**	2.34	8.56	−**25.05**
**Headache**										
No	285	0.05	**17.26**	1.86	0.00	**0.36**	0.03	−1.79	**26.93**	7.79
Mild	1,082	**3.28**	0.62	0.84	**0.18**	0.02	0.03	−**19.12**	−7.00	7.15
Moderate	471	1.33	**2.64**	**4.68**	0.04	0.06	**0.09**	9.44	−**11.16**	−**13.10**
Severe	157	**9.40**	0.51	0.24	**0.26**	0.01	0.00	**22.81**	−4.45	−2.70
**Vomiting**										
No	1,769	0.60	0.11	1.58	0.13	0.02	0.20	−16.37	5.99	19.75
Yes	226	**4.66**	0.88	**12.35**	0.13	0.02	**0.20**	**16.37**	−5.99	−**19.75**
**Shivering**										
No	771	1.77	0.57	**8.96**	0.07	0.02	**0.20**	−12.14	−5.81	**20.22**
Yes	1,224	1.12	0.36	**5.64**	0.07	0.02	**0.20**	12.14	5.81	−**20.22**
**Diarrhoea**										
No	1,809	0.06	0.19	1.05	0.02	0.04	0.16	−5.54	8.61	17.76
Yes	186	0.55	1.87	**10.22**	0.02	0.04	**0.16**	5.54	−8.61	−**17.76**
**Urine colour**										
Amber	982	0.07	**3.92**	1.12	0.00	**0.14**	0.03	−2.62	**16.68**	7.87
Brown	104	0.16	1.48	1.70	0.00	0.03	0.03	2.95	−7.50	7.09
Normal	909	0.02	**2.71**	**2.38**	0.00	**0.09**	0.06	1.31	−**13.40**	−**11.06**
**Abdominal pain**										
No	1,066	0.03	**9.95**	0.05	0.00	**0.39**	0.00	1.89	**27.74**	−1.67
Yes	929	0.04	**11.42**	0.05	0.00	**0.39**	0.00	−1.89	−**27.74**	1.67
**Rash**										
No	1,933	0.05	0.02	0.01	0.04	0.01	0.00	−9.32	4.99	2.19
Yes	62	1.65	0.67	0.17	0.04	0.01	0.00	9.32	−4.99	−2.19
**Previous bouts of malaria**										
No	1,034	0.99	**8.02**	0.03	0.05	**0.30**	0.00	10.25	**24.49**	1.37
Yes	961	1.07	**8.63**	0.03	0.05	**0.30**	0.00	−10.25	−**24.49**	−1.37

Values in bold show the axis on which each modality contributed (contribution value greater than 2.5 indicates a contribution) and where it had greater quality representation (cosine squared). ≤ −2 or ≥ 2 (values in bold) were taken as cut-off points for the test values for significant representation. Modality consists of variables associated with a specific pole for each profile, as identified by the test value sign (negative or positive).

**Table 4:** Test values for illustrative variables.

Supplementary variables	n	Test values		
		Axis 1	Axis 2	Axis 3
***P. vivax*** **infection**				
No	583	−0.57	**8.07**	**3.39**
Yes	1,412	0.57	−**8.07**	−**3.39**
***P. falciparum*** **infection**				
No	1,563	**7.23**	**2.11**	−1.41
Yes	432	−**7.23**	−**2.11**	1.41
***P. malariae*** **infection**				
No	1,133	−0.70	**3.16**	**2.38**
Yes	862	0.70	−**3.16**	−**2.38**
**Parasitaemia**				
1–1,999	177	0.85	−1.49	−0.87
2,000–4,999	159	**4.58**	−1.76	−**3.73**
5,000–9,999	145	**2.10**	−0.28	−**2.30**
>9,999	256	**4.27**	0.45	−**6.25**

≤−2 or ≥ 2 (values in bold) were taken as cut-off points for the test values for significant representation. Modality consists of variables associated with a specific pole for each profile, as identified by the test value sign (negative or positive).

**Table 5:** Profile structure.

**Pole**	**Profile**		
	**1**	**2**	**3**
**Negative**	Puerto Nariño	Abdominal pain, yes	Fever, yes
	Area 4	Previous bouts of malaria, yes	Shivering, yes
	Headache, mild	Stagnant water, no	Vomiting, yes
	Stagnant water, no	19–60 years of age	Diarrhoea, yes
	Shivering, no	Normal-coloured urine	Puerto Nariño
	Previous malaria, yes	Headache, moderate	Headache, moderate
	***P. falciparum***	Diarrhoea, yes	Normal-coloured urine
	**Parasitaemia 2,000–4,999**	Fever, no	Area 4
		Headache, mild	***P. vivax***
		***P. falciparum***	***P. malariae***
		***P. malariae***	**Parasitaemia >9,999**
		***P. vivax***	**Parasitaemia 2,000–4,999**
**Positive**	Headache, moderate	Fever, yes	Headache, no
	Previous bouts of malaria, no	Amber-coloured urine	Amber-coloured urine
	Mosquito net use, no	≤5 years	Area 2
	Shivering, yes	Water stagnation, yes	Area 1
	Vomiting, yes	Previous bouts of malaria, no	19–60 years of age
	Stagnant water, yes	Headache, no	Leticia
	Insecticide use, yes	Abdominal pain, no	Shivering, no
	Headache, severe		Fever, no
	Area 1		
	Leticia		

Modality consists of the variables making up each profile (negative and positive poles), taking the contribution, cosine squared and test values into account. Illustrative variables enriching each profile are indicated in bold.

The first profile consisted of variables related to the area in which the patients reside, their sanitary conditions (i.e. nearby stagnant water, mosquito nets and insecticide use) and certain symptoms (i.e. headache, shivering and vomiting). Residing in Puerto Nariño, area 4, having no stagnant water nearby, having a history of malaria and displaying mild symptoms (i.e. mild headache without shivering) correlated with *P. falciparum* infection (Table 5 and Fig. 4a).

The second profile related to triple *P. falciparum, P. vivax* and *P. malariae* infection, and the variables covered age, living conditions, medical history and symptoms (i.e. 19–60 years of age, having no stagnant water nearby, having a history of malaria, symptoms including abdominal pain, normal-coloured urine, slight to moderate headache, diarrhoea and no fever) (Table 5 and Fig. 4b).

The third profile (double *P. vivax* and *P. malariae* infection) highlighted more severe symptomatology, together with parasitaemia. The variables included living in Puerto Nariño, area 4, fever, shivering, vomiting, diarrhoea, moderate headache, normal-coloured urine and >9,999 or between 2,000–4,999 parasitaemia (Table 5 and Fig. 4c).

## Discussion

The climatic, environmental and geographic characteristics in South America provide favourable conditions for the circulation of *Plasmodium* spp. and vector-borne diseases such as malaria, which thereby poses a significant threat to public health in countries such as Colombia^22^. The population living in the Colombian Amazon region is particularly vulnerable, showing high malarial morbidity and mortality^7,9^.

In this report, most of the study population resided in rural areas lacking access to water, sewerage and/or gas (i.e. public services). Their type of housing is palafitic (i.e. stilt houses over water/alongside a river supported by pillars or simple stakes, or houses built on bodies of calm water such as lakes, lagoons and slow running large rivers), often but not always having palm-leaf roofs and wooden walls, thereby exposing their inhabitants to the environment and the vector’s ecosystem, thus increasing their probability of acquiring parasitic infections^23^. Such living conditions result in the high prevalence of malaria in this population, and in other populations in Colombia and worldwide^8,24,25^.

The active search for parasite infections has involved the simultaneous use of molecular and conventional microscopy techniques. This approach has enabled the diagnosis of *P. malariae* and mixed-species malaria infections (Additional file 2: Fig. S1). TBS as a diagnostic tool for malaria may not be sufficient as it leads to under-reporting (mainly of mixed-species malaria) and is limited in its ability of ensure timely treatment. Its use must thus be complemented by techniques providing greater sensitivity (i.e. molecular techniques)^10,13,26,27^. Prompt and accurate diagnosis of malarial infection in symptomatic populations and the identification of asymptomatic and sub-microscopic infections contributing to transmission can thus constitute part of the effective control and management of disease, with a view to eliminating malaria^28,29^.

Using molecular techniques enabled the identification of a large number of parasite infections and a high PDI (Additional file 6: Table S3) for Colombia; municipalities in Colombia’s Pacific region and the Antioquia region have reported similar results in terms of infection and PDI^30^. Molecular diagnostic tools have enabled the successful and highly sensitive detection of parasite species involved in mixed infections. In this study, more than 40% of the target population had mixed-species infections (Fig. 3 and Additional file 2: Fig. S1b), which was consistent with previous reports in India^20^, Thailand^31^, Papua New Guinea^32^ and Brazil^33^.

As previously reported for Colombia, *P. vivax* was associated with the highest frequency of malaria in all localities evaluated (Fig. 2)^3,34^; conversely, in the Peruvian Amazonian region the prevalence of this species varies in accordance with the area being evaluated^35^.

*P. malariae* was the second most highly ranked species in terms of disease frequency and contribution to infections (Fig. 2). This parasite species is known to be widespread across sub-Saharan Africa and south-eastern Asia^36^; however, molecular detection methods identified a higher proportion of *P. malariae* compared with microscopy in our study and in previous studies in Colombia and worldwide^3,10,31,33^.

*P. falciparum* showed lower prevalence and contribution to cases of malaria in the target population. A differential infection frequency was detected for this species with respect to the type of settlement, with the number of cases of infection with this parasite being greater in rural populations (Fig. 4b and Additional file 6: Table S3).

Differential parasitaemia levels were detected amongst the different areas being sampled. Individuals living in endemic areas who have been exposed to the parasite from an early age display a certain degree of immunity, as exemplified by low parasitaemia levels when exposed to new infections^37,38^. This may partially explain why the population inhabiting area 4 had the lowest levels of parasitaemia (Additional file 4: Fig S3b), consistent with the fact that more than 50% of this area’s inhabitants had suffered previous episodes of malaria. However, further studies regarding the association between previous episodes of malaria and parasitaemia levels are needed.

Evaluating the factors associated with mixed-species infection revealed that high parasitaemia levels were less frequently associated with simultaneous *P. falciparum* and *P. malariae* infection (Table 2 and Additional file 9: Table S4). Cross protection has been reported for these two parasite species^39^, as they share common antigens^37,40^, therefore host immunity limits parasitaemia in this type of mixed infection.

Parasite infection may be favoured by certain host characteristics that increase the interaction of parasites with target cells, thereby leading to greater infection success^41,42^; for example, the probability of being bitten and the transmission frequency is greater in endemic populations^32,43,44^. Some areas within the Colombian Amazon region were found to be associated with higher levels of parasite infection; mixed infections (*P. vivax* and *P. malariae*) were associated with localities in areas 1 and 4 and Puerto Nariño (Additional file 9: Tables S4 and 5), whereas *P. falciparum* infection was concentrated in rural populations, mainly in localities in area 4 (Table 5 and Additional file 6: Table S3).

Spatial factors influence the parasite-host-vector interaction and contribute towards the appearance of high transmission foci or hotspots within a geographical area^45,46^. In these foci, high levels of parasite circulation are observed, thereby facilitating dispersion to other localities and contributing to the spread of infections^47,48^.

The mean parasitaemia values were similar for different types of infection (single or mixed) (Additional file 4: Fig. S3a), suggesting that more than one species of the same organism did not seem to have an additive effect on the amount of circulating parasites. Previous studies have proposed a density-dependent regulation mechanism interacting with other factors such as a species-genotype specific immune response, resulting in stabilisation of the *Plasmodium* population and episodes that are not dependent on infection by particular species^49^, which may help to explain our findings.

The coexistence of more than one parasite species in the same individual may be mediated by host and pathogen factors, such as the host immune response initially directed against the species or genotype at the highest density, thereby favouring the persistence of infection at lower density in a particular host^39^. The species/genotype coexistence model is controlled by parasite density-dependent regulation mechanisms; this model suggests that parasitaemia of the first infecting species (which has the highest prevalence amongst the target population) is downregulated on coinfection with the second species (which has the lowest prevalence). However, when the most prevalent species exceeds a threshold, the hosts’ immune response is triggered to limit the infection; such a mechanism is turned off once the parasite density is under control, thereby favouring population growth of the second species in mixed infections and persistence of the parasites in the host^39,44,49^.

Such mechanisms are largely modulated by the host. Our study evaluated whether specific clinical profiles amongst the target population were linked to infection with particular *Plasmodium* species. Fever was the symptom detected at the greatest frequency with all parasite species (Additional file 8: Fig. S5), as well as headaches for mixed infections (Additional file 7: Fig. S4).

MCA revealed dependent relationships between active and illustrative variables (Tables 3 and 4) and three profiles were compiled from the results (Table 5 and Fig. 4). The first profile suggested that symptoms such as headache and diarrhoea, along with previous episodes of malaria, occurred in the target population regardless of the species or infection status (single or mixed). It has been reported that infection-derived immunity in regions with constant parasite circulation (endemic regions), such as the Colombian Amazon region, induces a clinical course with non-specific symptomatology^25^.

The second profile related to triple infection and a population aged from 19 to 60 years (Fig. 4b and Table 5). High mixed infection frequencies were observed in this age group (Additional file 3: Fig. S2a), i.e. the economically-active population who are potentially those most exposed to mosquito bites and therefore to parasite transmission. The target region’s economic activity is related to artisan-produced handicrafts exploiting wood, fishing, mining and small-scale cultivation in community gardens, all situations that favour the transmission of disease and limit the effectiveness of parasite control measures^22,34^.

The third profile related to severe symptoms (i.e. fever) and mixed *P. vivax* and *P. malariae* infections (Fig. 4c and Table 5). This profile supported the aforementioned parasite density-dependent population regulation model^39,49,50^. This model illustrates that a parasite species present at higher density would influence the growth of other parasite species activating typical clinical symptoms in the host and maintaining stability of the population dynamics of parasite species^51^.

In-depth analysis is required for defining infection hotspots. Time series analysis should be used for parasite detection to establish whether infection events are due to transient infection or transmission foci, and risk maps and the population distribution (for host and vector) should be analysed to determine the localities of disease cases^16,46,48^. Identifying whether a specific area has high disease transmission enables appropriate management strategies to be designed to effectively limit the parasite’s transmission cycle^47,48^.

Control measures implemented in Colombia have focused on reducing the disease burden by the large-scale provision of insecticide-treated mosquito nets, periodic intra-household spraying and the presence of government agencies responsible for control, diagnosis and treatment^12,30,52^. Although endemic countries have introduced disease mitigation measures, they have not had the desired impact as the number of malaria cases has increased, particularly in rural areas^7^.

The present study actively searched for symptomatic patients in geographically isolated localities lacking nearby healthcare posts. The average family income is less than $250 per month in these areas, so a trip to a health centre represents a considerable family expense (around $50 per trip), so many parasitic infections are not seen or treated by healthcare control programmes^22^.

Greater malaria control efforts are required for progression towards the elimination of this disease; thus, understanding the distribution patterns of particular parasite species and the factors that influence malaria transmission in the Colombian Amazon region is crucial. The results of this study provide additional insight into malarial infections in the Colombian Amazon region, helping define the areas to be prioritised in terms of malaria prevention and control measures, with the aim of decreasing malarial incidence and approaching the long-term goal of eradication.

## Methods

### Study area and population

This transversal study was carried out from July 2015 to April 2016; it included the population of the Colombian Amazon trapezium, inhabitants from the towns of Leticia and Puerto Nariño and rural settlements located along the banks of the Amazon and Loretuyacu rivers. The Colombian Amazon region represents 42% of Colombia’s territory and is formed by the Caquetá, Putumayo, Vaupés, Guainía, Guaviare and Amazon departments, the latter comprising the greatest geographical area^12,53,54^. The Amazon department has 77,088 inhabitants (population density: 1.5 inhabitants per km^2^)^12^. The town of Leticia and its surrounding communities had a projected population of 41,639 inhabitants according to the Departamento Administrativo Nacional de Estadística (DANE – Colombian Official Statistics Department) 2016 figures; Puerto Nariño and its neighbouring communities accounted for 8,279 inhabitants^12^.

Fifty-seven localities were sampled and grouped into four areas, taking into account their location and mobilisation towards basins converging on major tributaries (the Amazon and Loretuyacu rivers) (Fig. 1 and Additional file 1: Table S1). Area 1 included 32 localities (including the town of Leticia, the capital of the Amazon department and the remaining rural settlements), area 2 covered 10 localities (one settlement being mainly urban and the rest rural), area 3 covered seven localities (all rural) and area 4 covered eight localities (rural settlements all along the banks of the Loretuyacu river).

### Ethical considerations and sample-taking

Inclusion criteria consisted of recognising symptoms related to malarial infection when taking samples, such as headache, fever during the previous 8 days and sweating. People without malaria symptoms were not included in the study (exclusion criterion). The aim of the study was explained to patients; those who accepted an invitation to participate signed an informed consent form. A survey was then conducted that compiled information regarding participants’ socio-demographic characteristics and risk factors for malaria infection. This study was approved and supervised by the Universidad del Rosario’s (Colombia) School of Medicine and Health Sciences (EMCS) Research Ethics Committee (Comité de Ética en Investigacion - CEI) (CEI-ABN026-000161). Patients under 18 years of age who accepted the invitation to participate signed an informed consent, along with their tutors’ written approval. All methods and experiments were performed in accordance with the approved guidelines.

Two blood samples were collected simultaneously by capillary puncture. The first (TBS) was subjected to parasitological diagnosis by optical microscopy following Giemsa staining; the samples were processed and read on site at the time of sample collection. The second sample was stored on Flinders Technology Associates (FTA) cards and transported to the molecular biology laboratory of the FIDIC for molecular identification of the infecting parasite.

### Molecular diagnosis of *Plasmodium* spp

A Pure Link Genomic DNA mini kit (Invitrogen) was used for extracting the DNA from the FTA cards, following the manufacturer’s instructions. This was followed by PCR amplification of the extracted DNA to confirm the presence of the human *β-globin* constitutive gene segment^3,10^.

The infecting *Plasmodium* species (*P. vivax, P. falciparum* and/or *P. malariae*) were identified in the *β-globin*-positive samples by nested PCR. Specific primers against the 18 S rRNA fragment were used in the first round of PCR for genus detection and a second amplification (using the first PCR product as template) was performed to distinguish the *P. falciparum*, *P. vivax* and *P. malariae* species. The amplification conditions for these PCRs have been described previously by our group^3,10^.

### Statistical analysis

Descriptive statistics were used to summarise the sociodemographic variables, such as the sample-taking area, access to basic services (public water and electricity supply, sewerage service) and risk factors (nearby stagnant water, mosquito nets and insecticide use); these were presented as percentages with their respective 95% confidence intervals (95% CI). Age and parasitaemia (defined by TBS, as the number of parasites per 8,000 white cells/μL/number of white cells) were reported, along with their respective means and standard deviations (SD)^55^. The parasite density index (PDI) was taken as the amount of confirmed cases of malaria/population at risk^56^. Mixed infections were defined as the simultaneous detection of two or more *Plasmodium* spp. Fisher’s exact or Chi^2^ tests were used for establishing statistically significant differences amongst the data. ANOVA was used for comparing means and Bonferroni test was used for adjusting for multiple comparisons. A t-test was used for comparing the mean values for parasitaemia with the parasite infection status (single and/or mixed infection).

Logistic regression analysis was used for modelling the risk of a mixed infection, taking mixed infections as a dependent variable. Independent variables included in the model were age, residing in an urban or rural area, parasitaemia reported by TBS and housing conditions such as sewerage, gas and electricity supply, nearby stagnant water and mosquito net and insecticide use. STATA 12 software was used for analysing the data.

Multiple correspondence analysis (MCA) was used for establishing patient profiles, taking into account the nature of the clinical, epidemiological and laboratory variables (fundamentally categorical) estimated in this study. MCA was used for evaluating the degree to which each clinical and epidemiological variable participated in the compiling of profiles or groups of clinical significance in terms of similarity with or proximity to the different categories of variables, thus facilitating the incorporation of laboratory variables (infection presence/absence, parasitaemia) into these profiles’ for observing patterns^57–59^. In this way, groups were identified that had clinical significance from different groupings of categories of variables (i.e. this method was used to identify how sociodemographic characteristics and risk factors were grouped with single or multiple infections).

Two groups of variables were chosen for this analysis: active variables used for constructing factorial axes and supplementary or illustrative variables, which enriched factorial axes interpretation once they had been constructed^58^.

Sociodemographic, epidemiological and clinical variables were considered active variables, i.e. age, gender, origin, area, mosquito net and insecticide use, nearby stagnant water, fever, headache, vomiting, shivering, diarrhoea, urine colour, abdominal pain, outbreaks on the skin and previous episodes of malaria. The contribution values for each category were analysed for interpreting the axes compiled by the active variables, and the categories with a contribution value of more than 2.5 [mean contribution of 40 categories (100/40 = 2.5%)] were selected^57^.

Illustrative variables were the presence/absence of *P. vivax, P. falciparum* and *P. malariae* infection and parasitaemia. Cosine values were evaluated for estimating the quality of each active variable’s representation on each axis. The test values were used to determine whether the representation of each category on each axis significantly differed from 0 (≤−2 or ≥ 2 cut-off points), thus giving an evaluation of each category’s significance^57^.

The structure and formation of each profile were analysed using a bi-dimensional graphical representation. The active variables (epidemiological and clinical variables and risk factors) were represented on each axis by filled boxes and the nominal illustrative variables (infection by each of the three species and parasitaemia) were represented by empty rhombuses. The test values sign indicated each modality’s position on the positive or negative pole of each axis. Square size was proportional to each modality’s contribution on the most representative axis. Possible dependence and similarity relationships were identified, taking into account the distance between the variables represented on the graph, regarding the categories thus represented. SPAD-5 software was used for MCA.

## Electronic supplementary material

Supplementary information
