# Supplementary material for: Micro-epidemiology of mixed-species malaria infections in a rural population living in the Colombian Amazon region
Source: Sci Rep. 2018 Apr 3;8:5543. doi: 10.1038/s41598-018-23801-9 (PMC5883018; doi:10.1038/s41598-018-23801-9)
Supplement: Supplementary file 1 — Supplementary information [file 41598_2018_23801_MOESM1_ESM.pdf]

Micro-epidemiology of mixed-species malaria infection in a rural population living in the Colombian Amazon region

Milena Camargo [1,2†], Sara C. Soto-De León [1,3†], Luisa Del Río-Ospina [1†], Astrid C. Páez [1], Zanony González [1,5], Edgardo González [1], Juan R. Cubides [1], Paola A. Camargo-Ayala [1], Manuel E. Patarroyo [1,4], Manuel A. Patarroyo [1,6*]

[1] Molecular Biology and Immunology Department, Fundación Instituto de Inmunología de Colombia (FIDIC), Bogotá, Colombia

[2] PhD Programme in Biomedical and Biological Sciences, School of Medicine and Health Sciences, Universidad del Rosario, Bogotá DC, Colombia

[3] Universidad de Ciencias Aplicadas y Ambientales (UDCA), Bogotá, Colombia

[4] School of Medicine, Universidad Nacional de Colombia, Bogotá, Colombia

[5] Masters in Epidemiology Programme, School of Medicine and Health Sciences, Universidad del Rosario, Bogotá DC, Colombia

[6] Basic Sciences Department, School of Medicine and Health Sciences, Universidad del Rosario, Bogotá, Colombia

[†] These authors contributed equally to this work

* Corresponding author: Professor Manuel A. Patarroyo M.D., Dr.Sc.
Carrera 50#26-20, Bogotá, Colombia
e-mail: mapatarr.fidic@gmail.com (MAP)
Telephone number: (571) 3244672 ext. 142-143

Supplementary information legends

Additional file 1: Table S1. Description of the target locality and the areas and number of samples included in the study.

Additional file 2: Fig. S1. Distribution of the relative frequencies of *Plasmodium* spp. infection and mixed infection, as determined by detection techniques (n=1,995). Part (**a**) describes thick blood smear (TBS) detection. Part (**b**) describes detection by PCR.

Additional file 3: Fig. S2. Parasite infection distribution/number and the correlation with age. Part (**a**) shows *Plasmodium* spp. infection (green line), mixed infections (white line). Part (**b**) shows *P. vivax* (red line), *P. malariae* (blue line) and *P. falciparum* infections (yellow line). The number of samples for each age group is shown in black.

Additional file 4: Fig. S3. Distribution of the means for parasitaemia. Part (**a**) compares the levels of parasitaemia for the areas sampled. Part (**b**) compares the levels of parasitaemia for single and multiple infections. The continuous line indicates the mean; higher and lower values are represented by whiskers; the dots represent extreme values. A statistically significant difference was observed between the mean parasitaemia values for areas 3 and 4 (Bonferroni test).

Additional file 5: Table S2. Distribution of infection cases for *Plasmodium* species among the localities/areas included in this study

Additional file 6: Table S3. Distribution of infection cases for *Plasmodium* species and the correlation with settlement type

Additional file 7: Fig. S4. Distribution of symptoms and the correlation with the *Plasmodium* spp. infection status, as determined by PCR. Blue represents the uninfected target population. Green represents that proportion of the target population infected by a single species. Dark red represents that proportion of the target population with a mixed infection.

Additional file 8: Fig. S5. Distribution of symptoms and the correlation with the infecting *Plasmodium* species

Additional file 9: Table S4. Logistic regression for modelling the relationship between risk factors and the combination of infecting species

Additional file 1: Table S1

Sampling Area	Locality	Acronym	Settlement Type	Number of Samples
Area1 (n=344)	Afasinte	AF	Rural	1
	Arara	AR	Rural	2
	Barrio Nuevo	BN	Rural	4
	Caballo Cocha	CC	Rural	1
	Canan	CA	Rural	4
	El Calderón	CL	Rural	1
	El Porvenir	PV	Rural	6
	Humarizal	HU	Rural	1
	Jardín	JA	Rural	1
	Jose Maria Hernandez	JH	Rural	1
	Km 11	K11	Rural	9
	Km 12	K12	Rural	1
	Km 18	K18	Rural	1
	Km 6	K6	Rural	3
	La Libertad	LL	Rural	1
	La Nueva Esperanza	NE	Rural	1
	La Playa	LP	Rural	2
	La Sarita	LS	Rural	2
	La Unión	UN	Rural	1
	Leticia	LT	Urban	182
	Nazareth	NZ	Rural	33
	Colombia	CO	Rural	1
	Puerto Triunfo	PT	Rural	1
	Punta Brava	PB	Rural	1
	San Antonio de los Lagos	SA	Rural	31
	San Juan Bosco	JB	Rural	1
	San Juan de los Parentes	JP	Rural	26
	Simon Bolivar	SB	Rural	1
	Ticoya	TC	Rural	8
	Victoria Negra	VN	Rural	2
	Yaguas	YA	Rural	2
	Zaragoza	ZA	Rural	12

Area 2 (n=257)	20 de Julio	JU	Rural	2
	El Vergel	VE	Rural	8
	Mocagua	MA	Rural	14
	Macedonia	MC	Rural	34
	Palmeras	PA	Rural	12
	Patrullero	PU	Rural	2
	Puerto Esperanza	PE	Rural	4
	Puerto Nariño	PN	Urban	15
	San Martin de Amacayacu	SM	Rural	164
	Valencia	VA	Rural	2
Area 3 (n=566)	12 de Octubre	OC	Rural	509
	7 de Agosto	AO	Rural	9
	Boyahuazú	BO	Rural	13
	Los Lagos	LA	Rural	4
	Naranjales	NA	Rural	5
	San Juan de Atacuari	SJ	Rural	25
	Tarapoto	TA	Rural	1
Area 4 (n=828)	Nuevo Paraíso	NP	Rural	8
	Puerto Rico	PR	Rural	87
	San Francisco	SF	Rural	13
	San Juan del Soco	JS	Rural	110
	San Pedro de Tipisca	SP	Rural	494
	Santa Teresita	ST	Rural	30
	Santarén	SR	Rural	65
	Villa Andrea	VI	Rural	21

Additional file 2: Fig. S1.

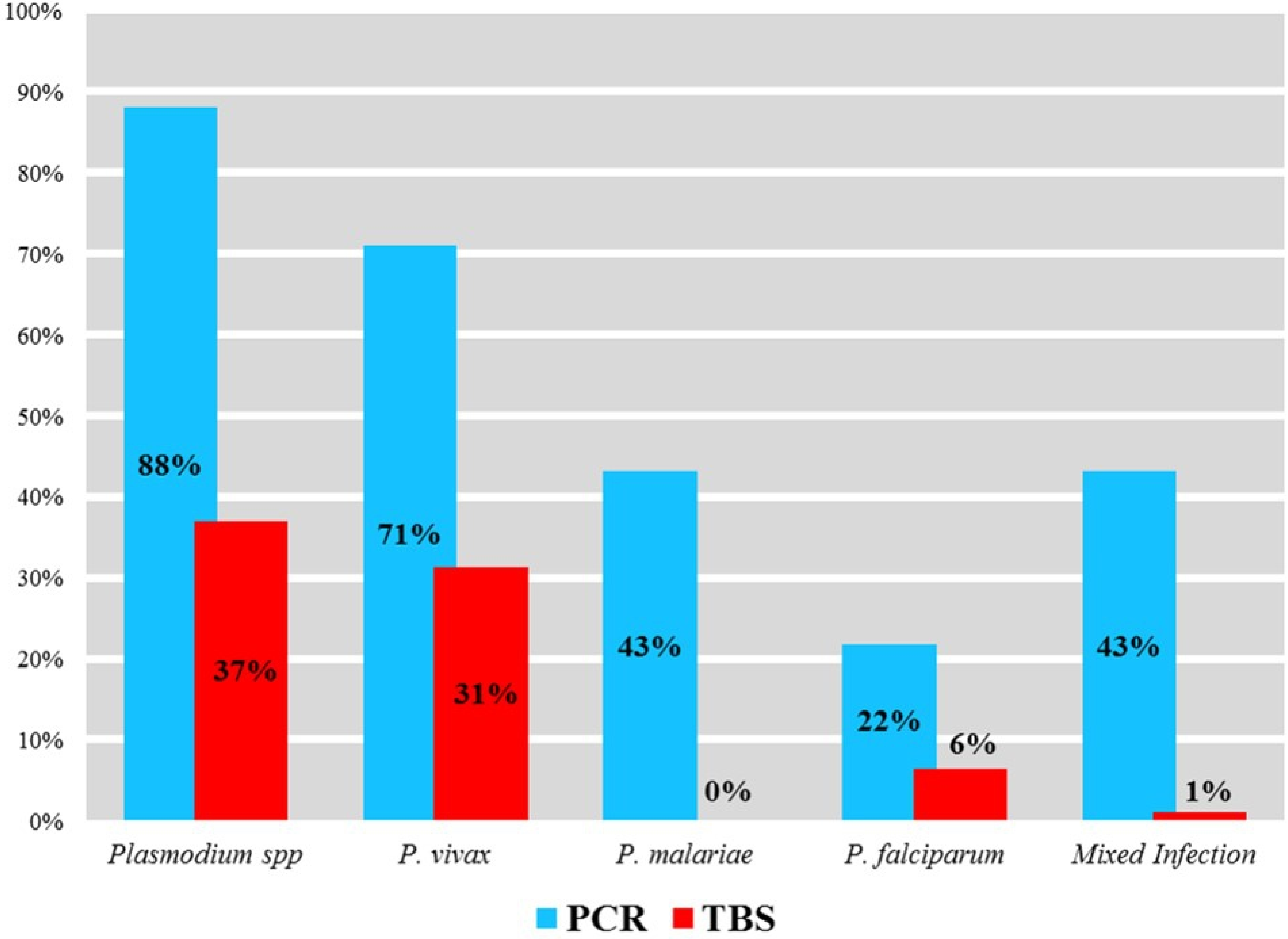

Additional file 3: Fig. S2.

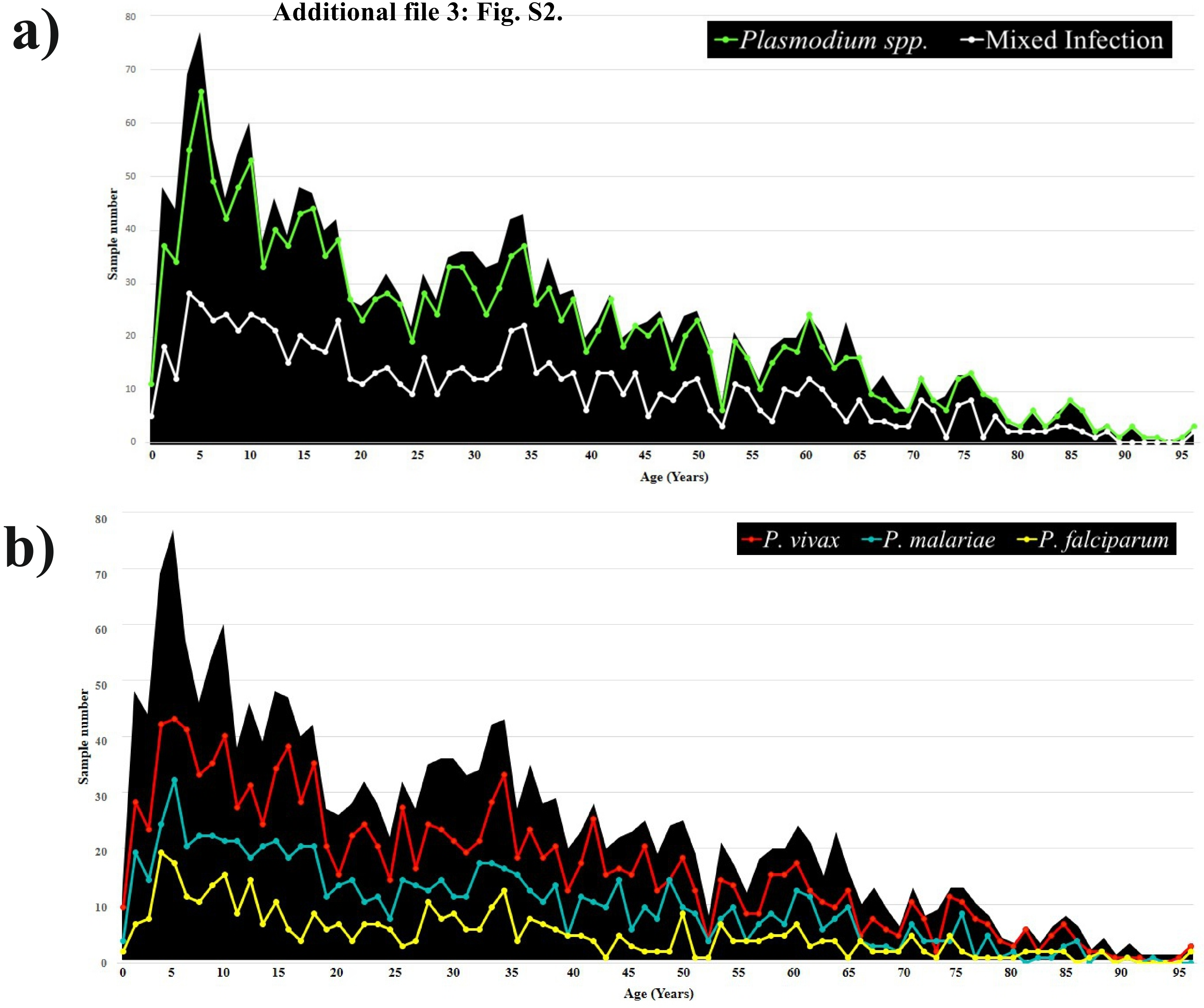

Additional file 4: Fig. S3.

a)

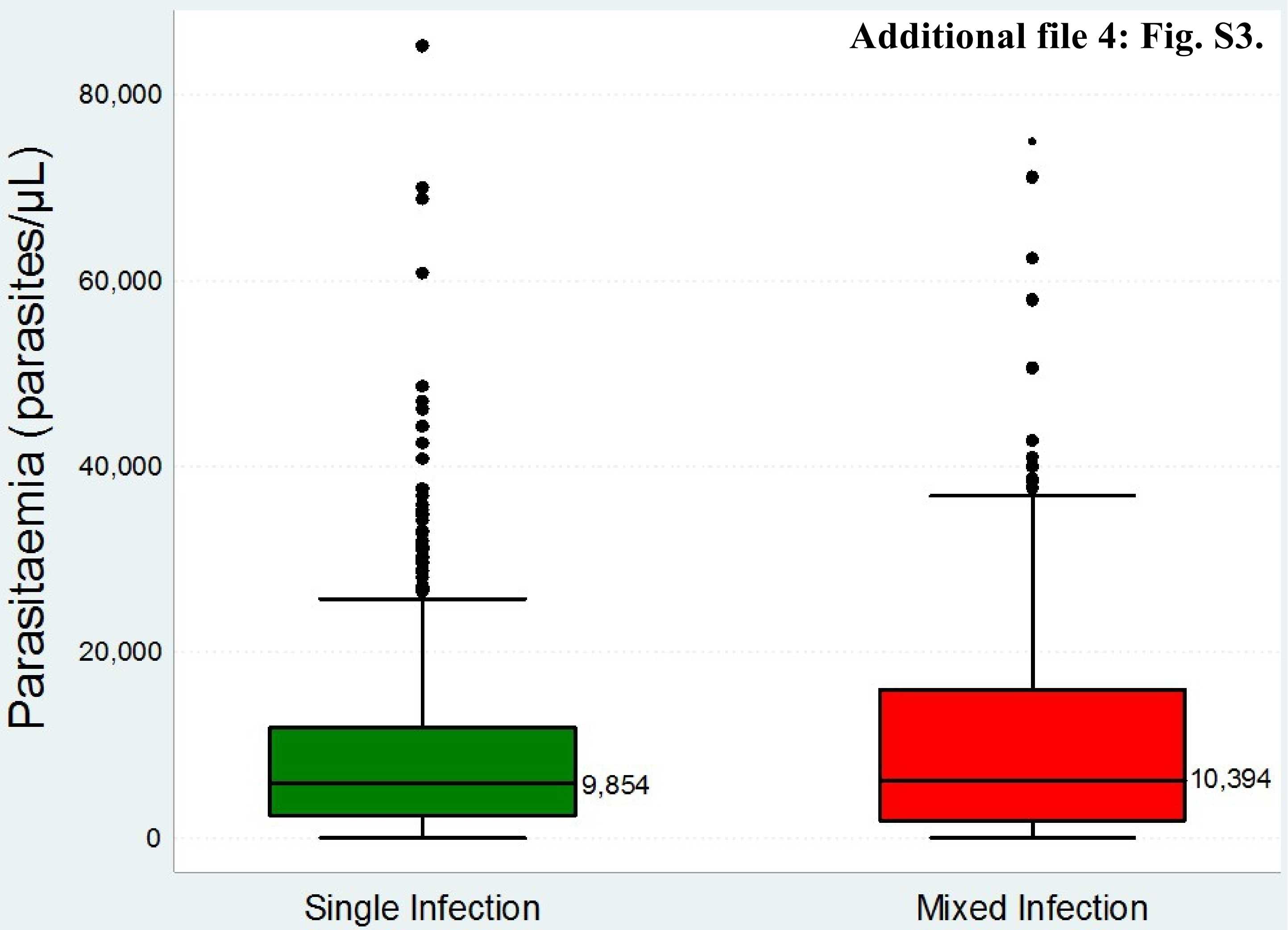

b)

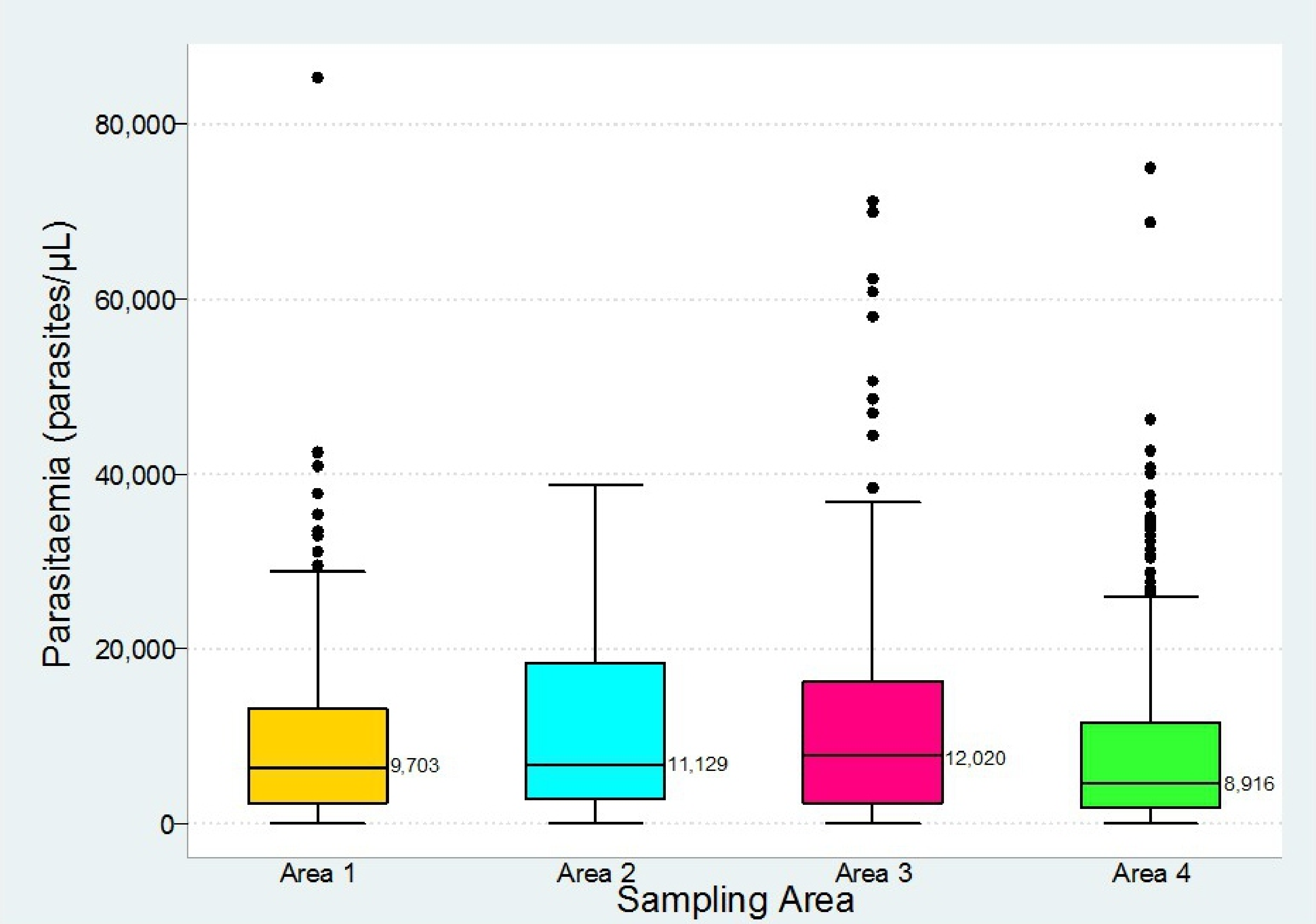

Additional file 5: Table S2.

**Locality** *Area 1*	Number of Samples	***Plasmodium spp.***		***P. vivax***		***P. malariae***		***P. falciparum***	
		n	%[†]	*n*	%[†]	*n*	%[†]	*n*	%[†]
Afasinte	1	1	100	1	100	1	100	0	0.0
Arara	2	2	100	2	100	0	0.0	0	0.0
Barrio Nuevo	4	4	100	4	100	2	50.0	0	0.0
Caballo Cocha	1	1	100	1	100	1	100	0	0.0
Canan	4	4	100	4	100	0	0.0	1	25.0
El Calderón	1	1	100	1	100	0	0.0	0	0.0
El Porvenir	6	6	100	4	66.7	2	33.3	0	0.0
Humarizal	1	1	100	1	100	1	100	0	0.0
Jardín	1	1	100	1	100	1	100	0	0.0
Jose Maria Hernandez	1	0	0.0	0	0.0	0	0.0	0	0.0
Km 11	9	9	100	6	66.7	9	100	0	0.0
Km 12	1	1	100	1	100	1	100	0	0.0
Km 18	1	1	100	1	100	0	0.0	0	0.0
Km 6	3	3	100	1	33.3	3	100	0	0.0
La Libertad	1	1	100	1	100	1	100	0	0.0
La Nueva Esperanza	1	1	100	1	100	1	100	0	0.0
La Playa	2	1	50.0	1	50.0	1	50.0	0	0.0
La Sarita	2	1	50.0	1	50.0	1	50.0	0	0.0
La Unión	1	1	100	1	100	1	100	1	100
Leticia	182	166	91.2	153	84.1	74	40.7	8	4.4
Nazareth	33	26	78.8	20	60.6	22	66.7	0	0.0
Colombia	1	1	100	1	100	1	100	0	0.0
Puerto Triunfo	1	1	100	1	100	1	100	0	0.0
Punta Brava	1	1	100	0	0.0	1	100	0	0.0
San Antonio de los Lagos	31	19	14	2	14	31	19	14	2
San Juan Bosco	1	1	100	1	100	0	0.0	0	0.0
San Juan de los Parentes	26	18	69.2	13	50.0	9	34.6	2	7.7
Simon Bolivar	1	0	0.0	0	0.0	0	0.0	0	0.0
Ticoya	8	8	100	6	75.0	5	62.5	3	37.5
Victoria Negra	2	1	50.0	1	50.0	0	0.0	0	0.0
Yaguas	2	2	100	0	0.0	1	50.0	1	50.0
Zaragoza	12	11	91.7	3	25.0	10	83.3	0	0.0

[†] Percentage are calculated taking into account the number of samples taken for each locality.

**Locality** *Area 2*	Number of Samples	***Plasmodium spp.***		***P. vivax***		***P. malariae***		***P. falciparum***	
		n	%†	*n*	%†	*n*	%†	*n*	%†
20 de Julio	2	2	100	2	100	0	0.0	0	0.0
El Vergel	8	8	100	7	87.5	4	50.0	0	0.0
Mocagua	14	14	100	14	100	5	35.7	3	21.4
Macedonia	34	25	73.5	15	44.1	15	44.1	9	26.5
Palmeras	12	9	75.0	8	66.7	5	41.7	3	25.0
Patrullero	2	2	100	1	50.0	1	50.0	0	0.0
Puerto Esperanza	4	4	100	4	100	2	50.0	2	50.0
Puerto Nariño	15	14	93.3	13	86.7	5	33.3	0	0.0
San Martin de Amacayacu	164	156	95.1	132	80.5	71	43.3	66	40.2
Valencia	2	2	100	1	50.0	1	50.0	1	50.0

† Percentage are calculated taking into account the number of samples taken for each locality.

**Locality** *Area 3*	Number of Samples	***Plasmodium spp.***		***P. vivax***		***P. malariae***		***P. falciparum***	
		n	%†	*n*	%†	*n*	%†	*n*	%†
12 de Octubre	509	450	88.4	364	71.5	230	45.2	110	21.6
7 de Agosto	9	9	100	7	77.8	5	55.6	1	11.1
Boyahuazú	13	11	84.6	10	76.9	4	30.8	0	0.0
Los Lagos	4	4	100	2	50.0	3	75.0	0	0.0
Naranjales	5	5	100	4	80.0	1	20.0	1	20.0
San Juan de Atacuari	25	24	96.0	16	64.0	8	32.0	11	44.0
Tarapoto	1	1	100	1	100	1	100	0	0.0

† Percentage are calculated taking into account the number of samples taken for each locality.

**Locality** *Area 4*	Number of Samples	***Plasmodium spp.***		***P. vivax***		***P. malariae***		***P. falciparum***	
		n	%†	*n*	%†	*n*	%†	*n*	%†
Nuevo Paraíso	8	6	75.0	3	37.5	4	50.0	3	37.5
Puerto Rico	87	81	93.1	66	75.9	48	55.2	21	24.1
San Francisco	13	10	76.9	9	69.2	5	38.5	0	0.0
San Juan del Soco	110	103	93.6	90	81.8	55	50.0	15	13.6
San Pedro de Tipisca	494	425	86.0	324	65.6	184	37.2	147	29.8
Santa Teresita	30	24	80.0	22	73.3	12	40.0	4	13.3
Santarén	65	47	72.3	36	55.4	21	32.3	12	18.5
Villa Andrea	21	19	90.5	15	71.4	8	38.1	5	23.8

† Percentage are calculated taking into account the number of samples taken for each locality.

Additional file 6: Table S3.

Locality	Number of Samples	*Plasmodium spp.*			*P. vivax*			*P. malariae*			*P. falciparum*		
	n	*n*	%+	PI°	*n*	%+	PI°	*n*	%+	PI°	*n*	%+	PI°
**Leticia**†	182	166	91.2	3.99	153	84.1	3.67	74	40.7	1.78	8	4.4	0.19
**Puerto Nariño**†	15	14	93.3	1.69	13	86.7	1.57	5	33.3	0.6	0	0.0	0
**Other Localities***	1,798	1,570	87.3	57.8	1,246	69.3	45.9	783	43.5	28.8	424	23.6	15.6
**Total**	1,995	1,750	87.7	22.7	1,412	70.8	18.3	862	43.2	11.2	432	21.7	5.6

PI= Parasite index; † Localities of Leticia and Puerto Nariño correspond to urban settlement type; * Other Localities correspond to rural settlement type; + Percentages were calculated taking into account the number of samples taken for each locality. ° PI was calculated taking into account the number of inhabitants projected in 2016 [1].

1 Carebilla, M. A. Plan de Desarrollo Amazonas 2016-2019. Gestión y Ejecución para el Bienestar, la Conservación Ambiental y la Paz. (2015).

Additional file 7: Fig. S4.

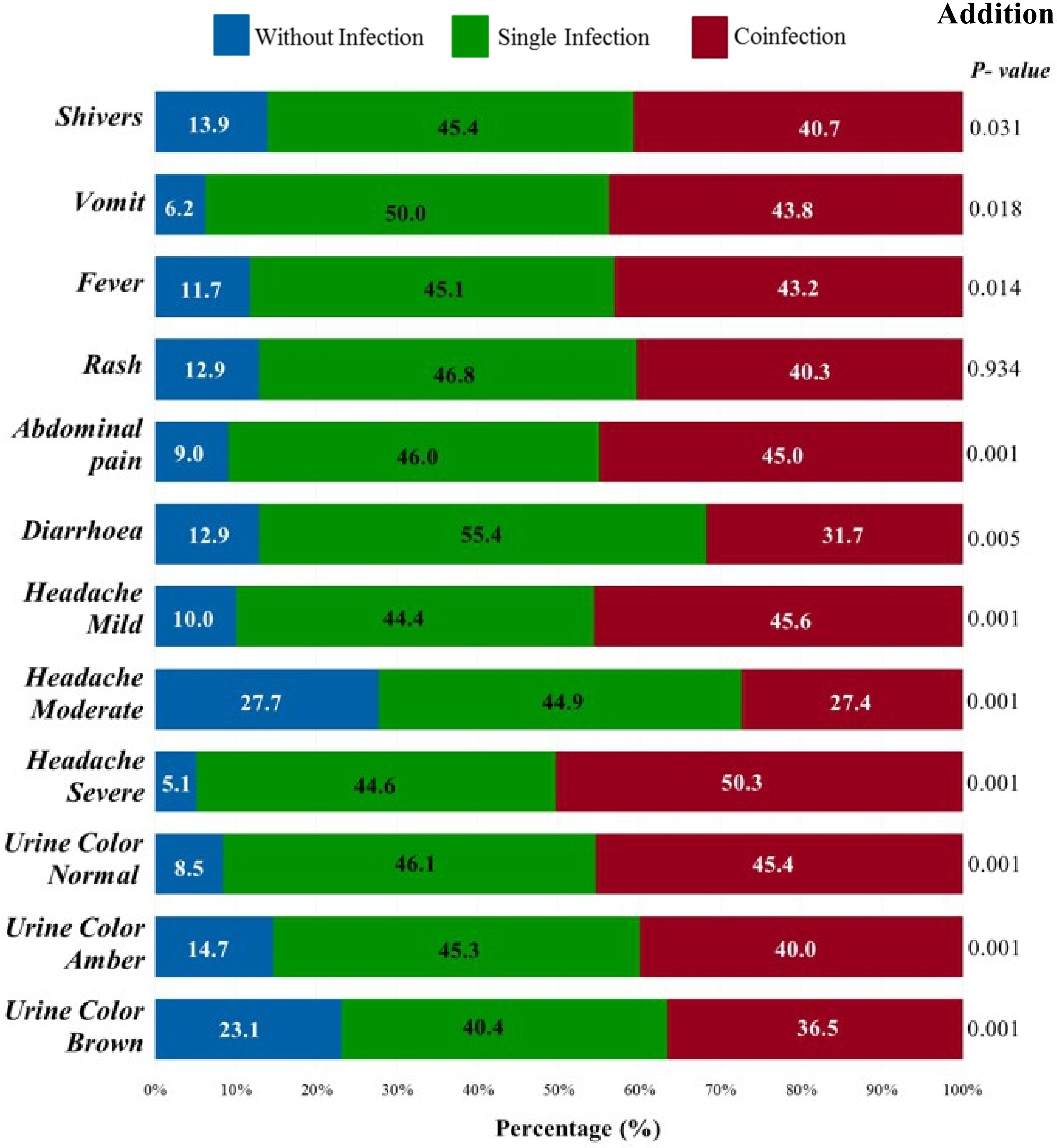

Additional file 8: Fig. S5.

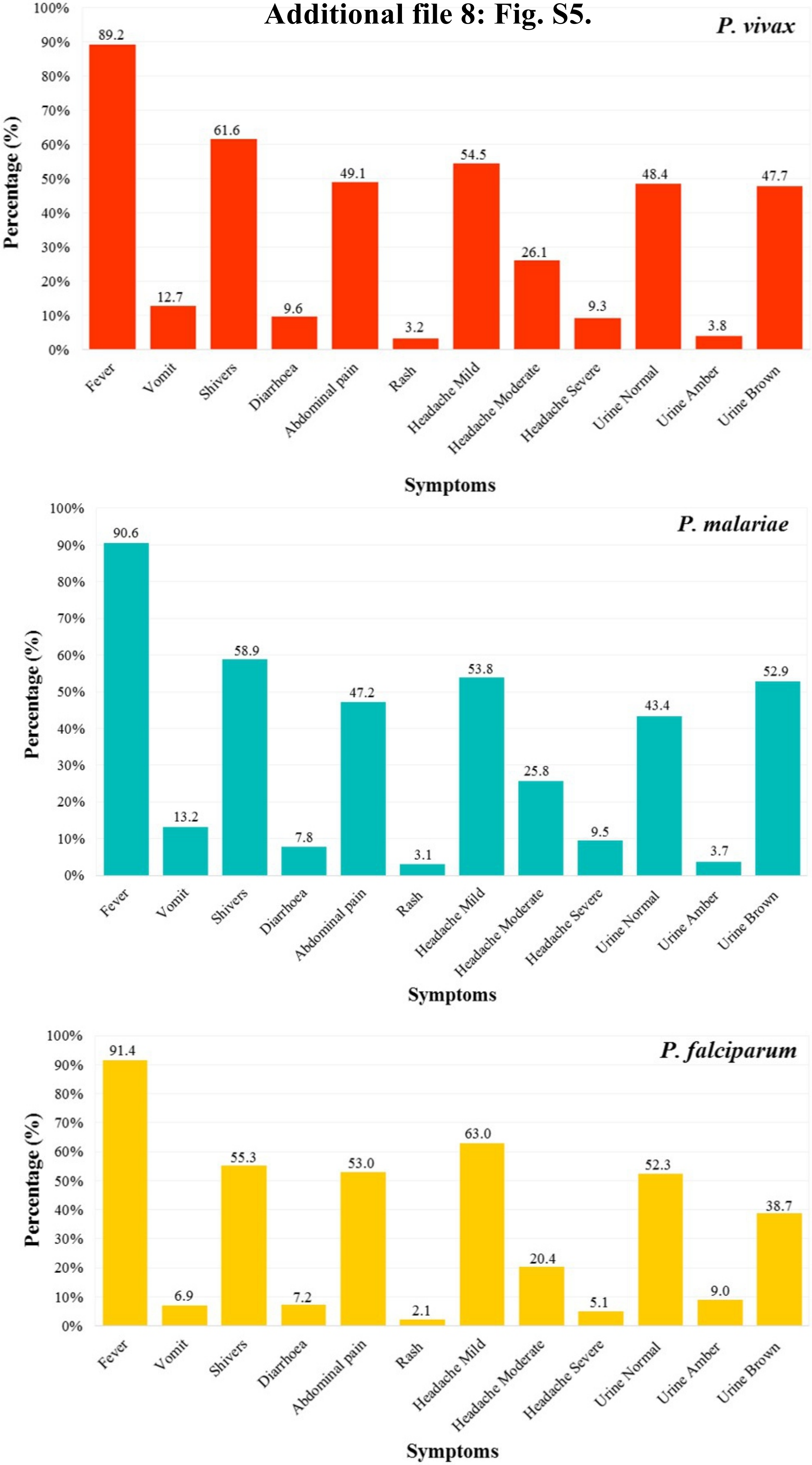

Additional file 9: Table S4.

	COMBINATION OF PARASITES SPECIES							
	P. vivax* and *P. malariae		***P. vivax* and *P. falciparum***		***P. malariae* and *P. falciparum***		***P. vivax* and *P. malariae* and *P. falciparum***	
**Variable**	**OR adjusted[a]**	**95%CI**	**OR adjusted[a]**	**95%CI**	**OR adjusted[a]**	**95%CI**	**OR adjusted[a]**	**95%CI**
**Age, years**								
≤5	0.87	0.48 - 1.58	1.15	0.40 - 3.27	0.56	0.10 - 3.00	1.16	0.40 - 3.34
6 - 12	0.88	0.43 – 1.77	0.82	0.33– 2.03	0.85	0.25 – 2.83	1.41	0.60 – 3.30
13 - 18	0.95	0.51 – 1.76	1.18	0.41 – 3.34	0.97	0.23 – 4.10	1.62	0.58 – 4.47
19 - 30	1.13	0.66 - 1.95	1.36	0.59 – 3.12	0.71	0.19 – 2.64	1.39	0.58 – 3.35
31 - 60	Reference		Reference		Reference		Reference	
≥60	0.41	0.16 - 1.00	1.80	0.56 – 5.71	0.76	0.47 - 1.23	1.03	0.21 – 4.99
**Gender**								
Male	Reference		Reference		Reference		Reference	
Female	1.00	0.71 - 1.40	0.86	0.47 - 1.56	1.84	0.71 – 4.72	1.59	0.86 – 2.96
**Sampling area**								
Area 1	**2.13**	**1.33 – 3.42**	0.29	0.83 – 1.05	0.77	0.43 – 1.39	0.88	0.20 – 1.67
Area 2	1.29	0.74 - 2.24	2.03	0.89 – 4.62	0.23	0.27 – 1.98	1.06	0.41 - 2.75
Area 3	0.66	0.18 – 2.45	1.43	0.29 – 7.10	0.83	0.52 – 1.34	0.78	0.98 – 1.65
Area 4	Reference		Reference		Reference		Reference	
**Water stagnation nearby**								
No	Reference		Reference		Reference		Reference	
Yes	1.11	0.77 - 1.60	0.85	0.44 - 1.61	1.42	0.52 – 3.88	0.72	0.36 - 1.41
**Insecticide use**								
No	Reference		Reference		Reference		Reference	
Yes	0.96	0.81 - 1.14	0.78	0.51 - 1.19	0.75	0.36 - 1.54	1.06	0.74 - 1.51
**Mosquito net use**								
No	Reference		Reference		Reference		Reference	
Yes	0.73	0.49- 1.12	0.58	0.18- 1.82	1.03	0.11- 8.96	3.32	0.41- 9.31
**Gas public service**								
No	Reference		Reference		Reference		Reference	
Yes	0.74	0.34 - 1.06	1.02	0.46 – 2.29	1.70	0.52 – 5.49	1.05	0.47 – 2.34
**Public light service**								
No	Reference		Reference		Reference		Reference	
Yes	0.60	0.49 - 1.57	2.05	0.58 – 7.20	1.78	0.20 - 9.89	1.48	0.41 – 5.27
**Public water**								
No	Reference		Reference		Reference		Reference	
Yes	0.88	0.70 - 2.42	0.93	0.30 - 2.85	0.90	0.98 - 2.28	1.05	0.20 – 2.74
**Sewer service**								
No	Reference		Reference		Reference		Reference	
Yes	1.33	0.29 - 1.56	0.52	0.15 - 1.77	1.90	0.54 – 2.69	2.34	0.12 – 3.92
**Fever**								
No	Reference		Reference		Reference		Reference	
Yes	0.67	0.95 - 1.85	0.50	1.71- 1.48	0.91	0.68 – 2.98	0.91	0.55 – 2.14
**Parasitaemia**								
1-1,999	1.55	0.99 – 2.44	0.67	0.31 – 1.44	0.49	0.16 – 1.47	0.43	0.17 – 1.04
2,000-4,999	0.93	0.55 – 1.58	0.78	0.33 – 1.82	0.61	0.17 – 2.16	**0.44**	**0.21 – 0.92**
5,000-9,999	0.94	0.56 – 1.57	0.63	0.25 – 1.57	**0.18**	**0.35 – 0.93**	**0.26**	**0.09 – 0.68**
>9,999	Reference		Reference		Reference		Reference	

Values in bold= $p<0.05$

[a]OR adjusted for age, area, parasitaemia (thick blood smear detection), access to basic services (public water and electricity supply, sewerage service), nearby water stagnations, use of mosquito nets and use of insecticides.
